# When should genetic testing be performed in patients with neuroendocrine tumours?

**DOI:** 10.1007/s11154-017-9430-3

**Published:** 2017-09-30

**Authors:** Triona O’Shea, Maralyn Druce

**Affiliations:** Centre of Endocrinology, William Harvey Research Institute, Barts and the London School of Medicine and Dentistry, Queen Mary University of London, Charterhouse Square, London, EC1M 6BQ UK

**Keywords:** Neuroendocrine tumours, Phaeochromocytoma, Paraganglioma, Medullary thyroid cancer, Genetic screening

## Abstract

Neuroendocrine tumours (NETs) are a heterogenous group of tumours arising from neuroendocrine cells in several sites around the body. They include tumours of the gastroenteropancreatic system, phaeochromocytoma and paraganglioma and medullary thyroid cancer. In recent years, it has become increasingly apparent that a number of these tumours arise as a result of germline genetic mutations and are inherited in an autosomal dominant pattern. The number of genes implicated is increasing rapidly. Identifying which patients are likely to have a germline mutation enables clinicians to counsel patients adequately about their future disease risk, and allows for earlier detection of at-risk patients through family screening. The institution of screening and surveillance programmes may in turn lead to a major shift in presentation patterns for some of these tumours. In this review, we examine the features which may lead a clinician to suspect that a patient may have an inherited cause of a NET and we outline which underlying conditions should be suspected. We also discuss what type of screening may be appropriate in a variety of situations.

## Introduction

**Fig. 1:**
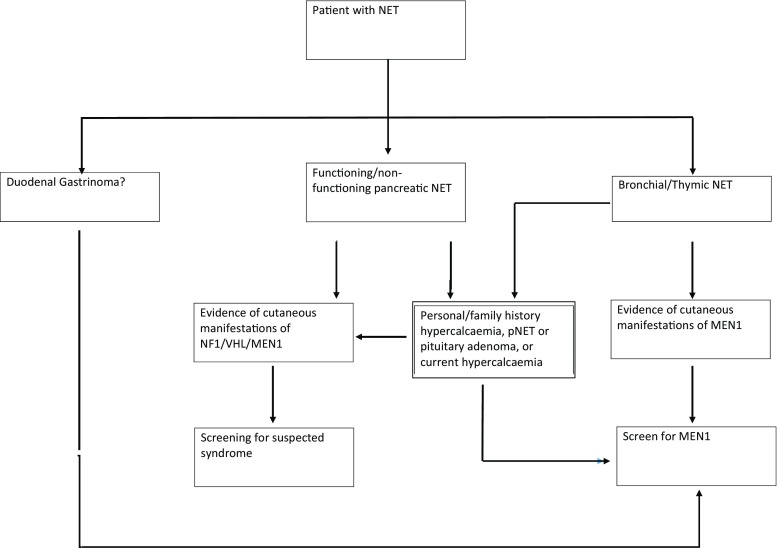
Proposed algorithm for genetic testing

Neuroendocrine tumours (NETs) arise from neuroendocrine cells - these cells produce neurotransmitters/neuromodulators, which are released from membrane-bound granules by exocytosis, in response to external stimuli. Message transmission occurs via the endocrine or paracrine route [1]. Neuroendocrine cells are distributed throughout the body as organs (hypothalamus, pituitary); tissues (adrenal medulla) and cells scattered within organs with other specialised functions (for example within the gastrointestinal tract or the lung). Neuroendocrine tumours may arise from any of these sites and may be secretory or non-secretory, leading to heterogeneous presentations. They include functioning and non-functioning gastroenteropancreatic tumours, catecholamine-secreting tumours, medullary thyroid cancer and chromophobe pituitary tumours [2] (Fig. 1).

NETs may present in a variety of ways depending on their site and functionality. They are frequently diagnosed incidentally when imaging is carried out for another reason; or following surgical resection of the appendix in a patient with appendicitis [3–5]. Local symptoms due to obstruction or mass effect cause another common mode of presentation, for example cough (bronchial NET) or small bowel obstruction (intestinal NET). Secretory syndromes such as the classical carcinoid syndrome of flushing and wheeze may also lead to the diagnosis in some cases. In addition, a number of these tumours are now known to be genetic in origin. Tumours may be detected in gene-positive patients who are under surveillance due to their increased risk of tumour development.

The more we understand about the genetic basis for these tumours, the more likely that they will be detected pre-clinically, in known gene carriers. However, the aim of our discussion here is to help the clinician faced with a patient with a newly-diagnosed NET, to decide whether or not genetic testing is warranted. We will focus on presentations of neuroendocrine tumours of the gastrointestinal system, chromaffin cell tumours (phaeochromocytoma and paraganglioma) and medullary thyroid cancer. Pituitary tumours have traditionally been treated as a separate group; however there is mounting interest in reclassifying them as Pituitary Neuroendocrine Tumours (PitNET) [6]. There is increasing evidence in the literature for a genetic predisposition for some subtypes of pituitary tumour, but discussion of this is beyond the scope of this review.

A wide range of germline genetic mutations are now known to be involved in the development of some NETs. Genetic screening if these conditions are suspected allows clinicians to counsel patients regarding future disease risk and allows for early detection of at-risk family members. It is also becoming increasingly clear that a genotype-phenotype relationship exists in many cases. The clearest example of this is in the case of medullary thyroid cancer (MTC), where although yield of genetic testing is only 25%, knowledge of the specific mutation alters management, with earlier surgery in those with a more aggressive gene mutation [7]. The same has not been shown definitively in other genetic syndromes such as MEN1, to date. However, in addition to determining which other conditions to seek in a patient who carries the MEN1 gene, there is emerging evidence that genotype may impact to an extent on the aggressiveness of the pancreatic NETs [8–10].

## Gastroenteropancreatic neuroendocrine Tumours (GEP-NETs)

GEP-NETs arise from the gastrointestinal tract, and historically were sub-classified by their embryological origin. Foregut NETs arise in the bronchial tree, stomach and pancreatic islet cells; midgut in the small intestine and hindgut in colon and rectum. As mentioned, these tumours may be incidentally found during imaging for other reasons, due to local pressure effect or due to symptoms as a result of hormone secretion [11]. In recent years nomenclature of GEP NETs has been revised, and they are now classified by the WHO by site and grade (as determined by Ki-67 score; which is a measure of the degree of expression of the protein Ki-67. This is expressed by proliferating cells, therefore a higher score suggests a higher rate of proliferation) [12–14]. Up to 10% of GEP NETs are estimated to have a hereditary background. Syndromes associated with these include multiple endocrine neoplasia 1 (MEN1), von Hippel Lindau (VHL), Neurofibromatosis Type 1 (NF1) and Tuberous Sclerosis (TS) [15], each of which are briefly outlined below, and summarised in Table 1.

**Table 1:** Gene mutations associated with tumours of the gastroenteropancreatic system

Gene	MEN1	RET	VHL	NF1
Location	11q13	10q11	3p26	17q11
Protein	Menin	Ret	VHL	Neurofibromin
Disease	MEN1	MEN2A	VHL	NF1
		MEN3		
		FMTC		
Endocrinopathies	Hyperparathyroidism Pituitary adenomas Gastrinomas Pancreatic NETCarcinoid tumours Adrenal adenomata Rarely phaeochromocytoma	MTC Phaeochromocytoma Hyperparathyroidism	Phaeochromocytoma Pancreatic NET (usually non-functioning)	Phaeochromocytoma Gastroenteropancreatic NET
Other Features	Lipomas Angiofibromas Collagenoma	MEN3- Marfanoid habitus Mucocutaneous neuromas Hirschprung’s Disease	Retinal Haemangioblastoma CNS Haemangioblastoma Clear cell renal cell carcinoma	Café-au-lait skin lesions Lisch nodules Axillary freckling Inguinal freckling Cutaneous neurofibromas

**Multiple endocrine neoplasia type 1 (MEN1)** is perhaps the best known of the endocrine tumour-prone syndromes. It is associated with a number of endocrine and non-endocrine tumours and it is inherited in an autosomal dominant pattern with a high penetrance (approaching 100% by age 50) [16]. Originally described by Wermer in 1967, who described a combination of gastrinoma, hyperparathyroidism and pituitary adenomas in a kindred [17], MEN1 is now know to be associated with around twenty endocrine and non-endocrine tumours, most frequently affecting the parathyroids, pituitary, pancreas, duodenum, adrenal cortex and more rarely lungs/thymic tissue [18]. MEN1 occurs due to mutations of the tumour suppressor *MEN1* gene [19]. This gene is located on chromosome 11q13 and encodes the protein *menin*. The majority of cases are associated with a truncated form of or absent menin protein [20]. The precise mechanisms by which menin acts as a tumour suppressor are unclear, but actions are thought to be tissue-specific [21]. Approximately 10–15% of all pNETs are associated with MEN1 and up to 80% of patients with MEN1 will develop pNETs [22]. The syndrome is frequently associated with functioning pancreatic NETs, which most commonly secrete gastrin, glucagon, insulin or pancreatic polypeptide; less frequently tumours which secrete vasoactive intestinal polypeptide or growth hormone releasing hormone [15]. Although MEN1 is frequently associated with gastrinomas, these are usually duodenal in origin [8].

**Von Hippel Lindau syndrome (VHL)** is an autosomal dominant condition, associated with mutations of the VHL gene. This gene is located on chromosome 3 and encodes a protein involved in the ubiquination and degredation of hypoxia-inducible-factor (HIF). The lack of HIF degradation drives overexpression of vascular-endothelial-growth factor F (VEGF) [23]. The commonest clinical features are retinal and central nervous system haemangioblastomas but it is also frequently associated with renal cell carcinomas, phaeochromocytomas and paragangliomas and occasionally with pancreatic neuroendocrine tumours [24].

**Neurofibromatosis Type 1 (NF1)** is characterised by cutaneous café-au-lait spots, Lisch nodules in the iris, freckling of the axillary and inguinal regions and multiple cutaneous neurofibromas. It is associated with phaeochromocytoma in approximately 1% of patients and GEP NETs in a similar percentage [15, 25]. NF1 is caused by a mutation in the tumour suppressor gene *NF1*, which is localised on the long arm of chromosome 17. It may function as a negative regulator of the *ras* oncogene signalling pathway. It is inherited in an autosomal dominant pattern with variable penetrance. Genetic testing is seldom required as the syndrome can usually be diagnosed on clinical grounds alone.

**Tuberous Sclerosis** is an autosomal dominant condition, which is caused by mutations of *TSC1* and *TSC 2* which encode for hamartin and tuberin proteins respectively [26]. The *TSC1-TSC2* complex act as tumour suppressors via suppression of the mTOR signalling pathway. It is characterised by multisystem hamartomatous lesions but has also been associated with islet cell tumours particularly insulinomas [15].

### The patient presenting with a pancreatic NET (pNET)

When a patient with other features of a tumour prone syndrome presents with a NET, relevant genetic testing should always be considered. For example, in a patient with previously-diagnosed hyperparathyroidism, MEN1 is a possible cause. For patients in whom a pancreatic NET is the first presentation of disease, the need for genetic testing should be considered carefully as the likelihood of a genetic cause being found is relatively small.

#### Non-functioning pancreatic NETs

The majority of pancreatic NETs are non-functioning and frequently present with symptoms due to local mass effect, or symptoms due to liver or other distant metastases [27]. The incidence of pancreatic NETs appears to be rising - this perceived increase is likely in part due to improved classification, better imaging techniques and incidental findings during imaging/endocopy as part of cancer screening [28]. MEN1 is frequently associated with non-functioning pancreatic NETs (pNETs), however the majority of non-functioning pNETs are not associated with an underlying mutation [28]. Non-hereditary NETs are usually solitary [15] and present at a later age [29]. Concurrent features such as hypercalcaemia or a previous history of renal stones should raise suspicion significantly. Tuberous sclerosis is occasionally associated with pancreatic neuroendocrine tumours which are usually non-functioning, but the NET is very unlikely to be the first presentation of this condition [26]. Patients with VHL have been found to have pNETs; reported frequency varies from 5 to 15% [24, 30] however again, other manifestations of the condition have almost certainly resulted in the diagnosis of VHL being made before the pNET is noted. Thus the associated pNETs are usually non-functioning and are almost never the presenting feature of the condition [31]. pNETs have been reported in patients with NF1 [15, 32] which are usually non-functional and although they are unlikely to be the first manifestation, the spectrum of features is wide and the clinical signs may be subtle. Careful full physical examination is therefore mandatory.

#### Functioning pancreatic NETs

Functioning tumours are more likely to present at an earlier stage due to the presence of characteristic symptoms depending on the tumour type. As with functioning pNETs, they may or may not be associated with a clinical syndrome [18]. Gastrinomas are rare tumours which arise in the pancreas or duodenum. The syndrome of hypergastrinaemia was first decribed by Zollinger and Ellison in 1955 [33]. Symptoms include gastro-oesophageal reflux, severe peptic ulcer disease and diarrhoea. The hallmark of Zollinger-Ellison syndrome (ZES) is hypergastrinaemia; the diagnosis can be made more difficult by the widespread use of proton pump inhibitors (PPI) which cause moderate hypergastrinaemia [34]. For this reason it is recommended that PPI therapy be stopped prior to measurement of gastrin, however in some patients with very severe symptoms this is inadvisable due to the risk of peptic ulceration [35]. Insulinomas are rare tumours arising from pancreatic islet cells. Patients present with symptoms of hypoglycaemia, and typically weight gain with or without associated acanthosis nigricans. Somatostatinomas are very rare tumours which may arise from the duodenum or pancreas, and are frequently asymptomatic, but may be associated with diabetes mellitus, gallstones and steatorrhoea [36]. Glucagonomas arise from the alpha cells of the pancreas and are associated with diabetes mellitus, insulin resistance and a characteristic rash (necrolytic migratory erythema) [37]. Tumours which secrete vasoactive intestinal peptide are associated with severe diarrhoea and electrolyte disturbances [37].

A variety of functioning GEP NETs are associated with MEN1, most frequently gastrinomas (in 40% of patients) or insulinomas (10%), less frequently VIPomas, somatostatinomas and glucagonomas [18]. Insulinomas and somatostinomas have been rarely reported in patients with NF1, as has the association of somatostatinoma with gastro-intestinal stromal tumour [32, 38, 39]. These have not been the presenting feature of NF1 in any patient, which has been diagnosed on the basis of the typical clinical phenotype.

### In which patient with pNETs should you suspect an underlying genetic cause?

#### Clinical features

In all patients with pNETS a thorough history and examination should be carried out, in particular looking for features of the syndromic associations of GEP-NETs; a skin examination may reveal lipomas/angiomas/lipomas/hypopigmented macules or gingival papules [40] (suggesting MEN1), or café-au-lait spots (suggesting NF1). Fundoscopy or slit lamp examination may reveal Lisch nodules (NF1) or retinal angiomas (VHL). If VHL is suspected following clinical examination then genetic testing can be carried out. A history of primary hyperparathyroidism or pituitary adenoma in patients with GEP-NET is strongly suggestive of MEN1, and genetic testing should be carried out to allow for extended family testing [41]; while a personal or family history of phaeochromocytoma/paraganglioma is suggestive of NF1 or VHL.

#### Family history

A diagnosis of MEN1 can be made in any NET in patients with a family history of MEN1. The diagnosis should be strongly suspected in patients with a family history of an MEN1 associated disease (primary hyperparathyroidism, pituitary adenoma, pancreatic NET, Zollinger-Ellison syndrome) or in those a family history of renal colic, early onset peptic-ulcer disease or sudden unexplained death in younger members of the family. In any patient presenting with a pNET at a young age (<40 years old) MEN1 should be considered [41]. Gastrinomas are the first presentation of MEN1 in 29–40% of patients; in most cases the patient will already have developed hyperparathyroidism which has not yet been diagnosed [42–44]. It is therefore reasonable to suggest that younger patients with a functioning or non-functioning pNET have screening for hyperparathyroidism and a pituitary profile to assess if there is any suggestion of other MEN1 associated tumours; genetic screening can follow if indicated. It is important to be cognisant of the fact that the genotype-phenotype correlation in MEN1 is not consistent even within a single family, and also that a significant minority (5–25%) of patients with MEN1 will not have an identifiable mutation within the *MEN1* gene [41].

#### Tumour type

##### Gastrinoma

Gastrinomas may arise in the pancreas or duodenum and are discussed in more detail when we consider NETs of the GI Tract. Pancreatic gastrinomas are usually single large sporadic tumours [45]; and while they may occur in patients with MEN1 they are seldom the cause of symptomatic Zollinger-Ellison syndrome in this cohort [46]. NF1 has rarely been associated with gastrinomas [47, 48]. However this autosomal dominant condition has a high penetrance and can usually be diagnosed due to the characteristic café-au-lait spots and bony dysplasia. There has been a single case report of pancreatic gastrinoma in association with NF2 [49]. As previously, we suggest that patients with a pancreatic gastrinoma have a thorough history and examination, with genetic testing arranged if clinically appropriate.

##### Insulinoma

Approximately 10% of patients with MEN1 will develop insulinomas [18, 19], and in these cases the tumours are more likely to be multiple and more likely to recur [50, 51]. On average insulinomas in patients with MEN1 present one decade earlier than sporadic cases [18]. Insulinoma appears to be more common in Asian patients with MEN1 [52]. Insulinoma is only occasionally the presenting feature of MEN1 [53–55]. As in the case of gastrinomas, patients will invariably have biochemical evidence of primary hyperparathyroidism at the time of presentation, even if this is not clinically apparent [55]. Thus, it is reasonable to suggest that all patients with insulinoma have screening for primary hyperparathyroidism, and that genetic screening be strongly considered in patients presenting at a younger age.

##### Other functioning NETs

Glucagonomas and VIPomas are rare pancreatic tumours, both of which have been associated with MEN1. We suggest a thorough clinical history and examination in patients with functioning NETs, with an emphasis on personal or family history suggestive of MEN1. In patients presenting at a young age, or with a suspicious family history genetic testing ought to be undertaken. In all patients, investigation for concurrent hyperparathyroidism should be carried out (and genetic screening if this is positive).

#### Tumour size and number

MEN1 associated pNETs usually present as multiple microadenomas, with some associated larger tumours which are frequently found incidentally or during screening [56]. MEN1 should be strongly suspected in patients with multiple small pNETs.

In summary, all patients with pancreatic NETs should undergo a detailed history and examination; paying particular attention to any features suggestive of an underlying syndrome (NF1, VHL, TS) and screening considered if these features are found. All patients with a family history of MEN1 associated conditions, all patients with multiple or duodenal as well as or in the absence of pancreatic gastrinomas and all patients presenting with insulinoma before the age of 40 should be strongly suspected to have MEN1 and screening be considered. In other patients with pancreatic NETs it is reasonable to suggest screening for hyperparathyroidism (through measurement of a serum calcium in the first instance, with parathyroid hormone measurement if this is elevated) and genetic screening performed if this is diagnosed.

### The patient presenting with a NET in the GI tract

NETs have been described throughout the GI tract. Approximately 1% of NETs arise in the stomach, 40–50% in the small intestine and 15% in the colon or rectum [57]. As with NETs elsewhere they may present due to symptoms of hormone excess if the tumour is functional (classically the typical carcinoid syndrome or Zollinger-Ellison syndrome), or due to local mass effect (frequently small bowel obstruction) [58].

#### In which patients presenting with a GI NET should you suspect an underlying genetic cause?

##### Tumour type

Gastrinoma

Duodenal gastrinomas occur in approximately 40% of patients with MEN1 [18] and about 25% of all patients with gastrinoma have MEN1 [19]. The duodenal tumours are usually small and multiple, and frequently metastasise to local lymph nodes, which is in contrast to tumours associated with sporadic disease which are more frequently pancreatic and consist of a single, relatively large adenoma [45]. The majority of patients with MEN1 will develop primary hyperparathyroidism by age 50 [18] and thus MEN1 should always be suspected in patients with gastrinoma and a personal or family history of hypercalcaemia or nephrolithiasis. Zollinger-Ellison syndrome may be the presenting feature of MEN1 in up to a third of patients [59], however the majority of these patients will have biochemical evidence of primary hyperparathyroidism at the time of presentation [35, 60]. Typically, patients with MEN1 associated gastrinoma present at a younger age than in sporadic cases [44, 61].

*Gastric NET* tumours are rare; but multiple, small gastric carcinoids (Type 2) may be found in over 70% of patients with MEN1 [61] but account for only 5% of all gastric NETs [62]. These are associated with ZES, and are thought to be as a result of persistent hypergastrinaemia; and are frequently found during investigation of this [63]. MEN1 should always be suspected if gastric carcinoids are found in the presence of ZES. NF1 has rarely been associated with gastrinomas [47, 48]. However this autosomal dominant condition has a high penetrance and can usually be diagnosed due to the characteristic café-au-lait spots and neurofibromas. There is a consensus that MEN1 should always be suspected in patients with ZES and another feature of MEN1 (hyperparathyroidism, pituitary adenoma, adrenal adenoma) or a family history of an MEN1 associated disease [64]. In 25% of patients with MEN1 there is no previous family history of the syndrome however [44, 60]. For this reason it is generally recommended that MEN1 screening be performed in all patients with ZES; in particular if the gastrin producing tumour is found in the duodenum [35].

Other small intestinal NETs

Duodenal somatostatin-producing tumours have also been reported in patients with MEN1 [65]. These are generally small and have been found incidentally during surgery for gastrinomas; they have not been associated with the somatostatinoma syndrome in this group [32]. Duodenal somatostatinomas have also been reported in patients with NF1, usually located in the peri-ampullary region [15, 32, 66]. In one series 14% of duodenal somatostatinomas were associated with NF1 [32] however in none of these cases was the somatostatinoma the presenting feature of NF1. More recently somatostatinomas have been reported in a small group of female patients with polycythaemia and paraganglioma; a somatic gain-of-function mutation in HIF2A was found in these patients [67, 68]. It is as yet unclear if this syndrome is confined to female patients [67, 68]. A case of polycythaemia and paraganglioma has been reported in a male adolescent patient with the same mutation, but had no evidence of somatostatinoma at the time of reporting [69].

Neuroendocrine tumours of the small bowel are rarely associated with familial syndromes, however a detailed family history remains important as a small number may be associated with a mutation in inositol polyphosphate multikinase [70–72]. It is likely that other cases are associated with genetic mutations which have not as yet been characterised. Given that only a very small proportion can be genetically explained currently, we cannot currently justify testing all patients with GI NETs. However, patients with a family history should be considered for testing for research purposes.

Rectal NETs

These tumours have not traditionally been associated with MEN1; however there is increasing evidence that a proportion of them may have a genetic basis. An association between rectal carcinoids and Lynch Syndrome has been reported [73] and there are case reports of rectal carcinoids being diagnosed in siblings [74, 75]. Therefore, as with small intestinal NETs we suggest a thorough family history, and consideration of genetic testing for research purposes.

## Bronchial and Thymic NETs

As with GEP NETs bronchial NETs may be functioning or non-functioning. Functioning tumours may present due to symptoms of the clinical syndrome (eg ectopic ACTH secretion, GHRH secretion, PTHrP secretion) [72, 76]. The classical “carcinoid syndrome” is seldom seen with bronchial NETs; its’ presence is usually suggestive of liver metastases [77]. Non-functioning tumours usually present due to cough, recurrent infections or haemoptysis [78]. The location of bronchial NETs is the most important predictor of symptoms; central tumours frequently produce symptoms while those located more peripherally are more frequently picked up incidentally when imaging for other reasons [79].Thymic carcinoids are frequently functional and may secrete a wide range of hormones; they are responsible for a significant proportion of cases of Cushing’s syndrome due to ectopic ACTH secretion [80] [81]. Less frequently they present due to mass effect [81].

### In which patients presenting with a bronchial/thymic NET should you suspect an underlying genetic cause?

Most bronchial/thymic NETs do not have an underlying genetic cause. Bronchial NETs are found in approximately 5% of patients with MEN1; whilst thymic carcinoids are found reported in 2–8% [41, 80, 82]. Conversely, approximately 25% of cases of bronchial/thymic NETs are associated with MEN1 [80]. Bronchial/thymic NET has been reported as the presenting feature of MEN1 in one series; however all patients had concurrent primary hyperparathyroidism and several had other MEN1 associated tumours [82]. Therefore, we suggest that MEN1 should be considered in patients with these tumours, and a similar approach taken to the patient with GEP NETs. All patients should have a detailed history and examination performed, paying particular attention to the family history. It is reasonable that all patients be evaluated for the existence of primary hyperparathyroidism, and young people also for biochemical screening for functional pituitary adenomas. If the family history is suggestive of MEN1; or another feature of MEN1 is found in the presenting patient then analysis of the *MEN1* gene should be carried out [79, 83].

## The patient with Phaeochromocytoma/Paraganglioma (PPGL)

These rare tumours arise from chromaffin cells of the adrenal medulla (phaeochromocytoma) or in extra-adrenal chromaffin cells of the sympathetic or parasympathetic system (paraganglioma). Classically they present with the triad of headache, palpitations and hypertension (sustained or paroxysmal) [84]. However a wide range of presentations have been described, with some patients presenting due to complications of catecholamine excess (cardiac complications, stroke, diabetes mellitus) whilst others are diagnosed following the incidental finding of an adrenal mass on imaging for other reasons [85]. Non-secretory PPGLs are often discovered incidentally, for example patients may present with a lump in the neck. Traditionally 10% of these tumours were thought to be due to underlying genetic syndromes, however this is now thought to be at least 40% [86]. Over the past two decades, our knowledge of the genetics of these tumours has expanded rapidly. A number of genetic syndromes are associated with PPGL including VHL, MEN2 and £ and NF1. Mutations in the succinate dehydrogenase genes (SDHA, SDHB, SDHC, SDHD, SDHAF2), which encode the mitochondrial enzyme succinate dehydrogenase which catalyses the oxidation of succinate to fumurate, and which is a key component of the Krebs cycle [87], are also associated with hereditary phaeochromocytoma/paraganglioma syndromes. As could be predicted inactivating mutations in fumarate hydratase produce a similar clinical spectrum of phaeochromocytoma/paraganglioma to that seen in SDH inactivation [88–90]. More recently mutations in the MYC-associated factor X gene (MAX), Endothelial pas domain protein 1/Hypoxia inducible factor type 2A (EPAS1/HIF2A) and TMEM127 have been found to be responsible for some cases of adrenal phaeochromocytoma and extra-adrenal thoracolumbar paraganglioma (~1.1% all cases) [67–69, 91]. These conditions are all inherited in an autosomal dominant manner, with variable degrees of penetrance. The main features of these are described in Table 2. HIF2A somatic mutations have been reported in patients with PPGL and polycythaemia; the exact mode of inheritance is unknown but it is likely to be a germline mosaic mutation [67–69].

**Table 2:** Germline mutations implicated in the development of phaeochromocytoma/paraganglioma (PPGL)

Gene	Location	PPGL syndrome	Secretory Pattern (all may be non-secretory)	Other Features
RET	10q11.21	Adrenal, frequently bilateral.	Adrenergic	MTC Primary Hyperparathyroidism MEN3- mucosal ganglioneuromas and marfanoid habitus
VHL	3p25.3	Commonly adrenal, frequently bilateral. Occasionally extra-adrenal, may be malignant	Noradrenergic	Retinal Haemangioblastoma CNS Haemangioblastoma Clear cell renal cell carcinoma Occasionally non-functioning pNET
NF1	17q11.2	Adrenal, may be bilateral	Adrenergic	Café-au-lait skin lesions Lisch nodules Axillary freckling Inguinal freckling Cutaneous neurofibromas GEP NET
SDHA	5p15.33	Rare, reports of extra-adrenal disease	Dopaminergic/Noradrenergic	Gastro-intestinal Stromal tumours (GIST) Leigh syndrome (rare)
SDHB	1p36.13	Adrenal or extra-adrenal disease (frequently abdominal), often malignant	Dopaminergic/Noradrenergic	Hamartomas Thyroid cancer GIST Renal cell carcinoma Rare reports pituitary adenoma
SDHC	1q23.3	Rare; reports of adrenal and extra-adrenal disease. Occasional reports malignancy.	Dopaminergic/Noradrenergic	GIST
SDHD	11q23.1	Frequently head and neck PGL, also associated with adrenal disease	Dopaminergic/Noradrenergic	Hamartomas Thyroid cancer GIST Renal cell carcinoma Rare reports pituitary adenoma
SDHAF2	11q12.2	Rare, reports of adrenal and extra-adrenal PPGL	Dopaminergic/Noradrenergic	Unknown
MAX	14q23.3	Adrenal disease or extra-adrenal reported. May be malignant.	Noradrenergic/Adrenergic	Unknown
TMEM127	2q11.2	Adrenal, rare reports of head and neck PGL	Adrenergic	Unknown

Given the relatively large number of genes implicated in the development of PPGL thus far, and the corresponding low yield of performing a blanket gene panel in all patients, a targeted approach to testing has been suggested by many authorities [92–99]. However with the advent of next generation screening, the gene panel approach is becoming increasingly cost effective and provides rapid results, with a recent study identifying driver mutations in 80% of PPGL tumours, although the clinical significance of every mutation is not yet known [100, 101]. With these limitations in mind we suggest a targeted approach to screening initially with consideration given to whole exome sequencing using next generation sequencing techniques in patients who are likely to have a genetic mutation not identified using a standard targeted gene panel.

### In which patients should we suspect a genetic cause of PPGL?

#### The patient with an adrenal Phaeochromocytoma

Mutations in a large number of genes are associated with adrenal phaeochromocytoma (RET, VHL, NF1, SDHA, SDHB, SDHC, SDHD, MAX, TMEM127), and most sporadic PPGLs arise in the adrenal glands [102]. However, a number of factors can point to a particular mutation. In the first instance, a detailed clinical history and examination should be undertaken, with a particular emphasis on family history. Careful clinical examination and family history is almost always sufficient in the diagnosis of NF1, as the characteristic features of the syndrome (described earlier) are invariably present. There have been reports of phaeochromocytoma as the presenting feature of NF1 [103, 104], but in these cases mild clinical features of the syndrome were present, although unnoticed previously. Similarly, the stigmata of VHL (also described above) are usually present at the time of presentation with phaechromocytoma. Family history of paraganglioma may suggest an SDH mutation [92].

A personal or family history of hyperparathyroidism, or particularly medullary thyroid cancer are very suggestive of MEN2 (traditionally known as MEN2A) in a patient with a phaeochromocytoma, as is sudden unexplained death at a young age in a family member. Bilateral disease is also a strong indicator of a genetic basis of disease [96]. Younger age (<45 years) at presentation is a strong predictor of germline mutations [96, 105, 106]. In particular, PPGL in the paediatric cohort are almost invariably associated with an underlying genetic abnormality; thus genetic testing should always be performed in these groups [107, 108].

Approximately one-third of malignant PPGLs in adults are associated with germline SDHB mutations [109, 110], and approximately 20% of patients with an identified SDHB mutation will develop a malignant PPGL [111]. There is also a high frequency of malignant PPGL in patients with SDHD mutations [111] although the malignant potential appears to be less than that of SDHB associated disease. Malignant PPGL have also been reported in patients with SDHC mutations, however the malignant potential is not yet clear [86, 112, 113]. A French-Canadian series recently reported that 30% of patients with a novel SDHC mutation had malignant PPGL [114]. VHL and MAX mutations are also known to be associated with metastatic disease. Given the high frequency of germline genetic mutations in patients with metastatic PPGL genetic testing should be performed in this group; in particular for SDHB mutations. Immunohistochemical staining of the resected tissue may also suggest the presence of an SDHB/SDHD mutation; as tumours affected by these mutations will have negative staining for SDHB antibodies [115]; this can be used to guide and streamline testing.

The pattern of catecholamine production has associated with the underlying mutation. Tumours associated with SDH mutations (of which SDHD mutations are more frequent in adrenal phaeochromocytoma) tend to be associated with elevated methoxytyramine and also normetanephrine; NF1 and MEN2 with elevated metanephrine and VHL with elevated normetanephrines [116]. Adrenergic PPGL are not associated with SDH mutations, even with metastastic disease [99, 117] although TMEM127 and MAX mutations are associated with adrenergic adrenal phaeochromocytoma [92]. Therefore, the secretory pattern of the PPGL can direct screening in these cases [115, 118].

In summary, in patients with adrenal phaeochromocytoma genetic screening should be considered in all and carried out in:

- Patients with a clinical examination or personal/family history suggestive of a syndromic cause/germline mutation- RET/VHL/NF1
- Patients <45 years old
- Those with malignant disease- SDHB, VHL, SDHD, SDHC, MAX
- Those with bilateral disease- MEN2/3, VHL, SDHB
- Those with predominantly dopaminergic or noradrenergic patterns of secretion- SDHB, SDHD, RET
- Those with negative staining of the tumour for SDHB antibodies

#### The patient with an extra-adrenal Paraganglioma

Extra-adrenal disease, as with malignant/bilateral disease is strongly associated with germline mutations [96, 119]. Abdominal/pelvic disease is most commonly associated with SDHB mutations [92]. Cardiac paragangliomas have been associated with a variety of SDH mutations [120]. Head and neck paragangliomas are associated with SDH mutations in approximately 50% of cases; most commonly SDHD (approximately 50%) but also with SDHB and SDHC mutations [106, 121]. Thus in head and neck PGL screening for an SDHD mutation could first be undertaken, followed by SDHB, SDHC and SDHA/AF2. Other extra-adrenal PGL are generally associated with SDHB/SDHD mutations, and less commonly with VHL, MAX or SDHC mutations [119, 120]. SDHD disease is usually maternally imprinted, therefore the pattern of transmission is down the paternal line, although rare cases of maternal inheritance have been described [122]. As with adrenal lesions, metastatic disease is most commonly associated with SDH mutations, in particular germline SDHB mutations [109, 110]. Patients with the Carney triad (paraganglioma, gastrointestinal stromal tumours and pulmonary chondroma) [123] can usually be identified due to the associated features; as yet no causative gene has been detected [124]. The familial association of pituitary adenomas with PPGLs (both adrenal and extra-adrenal) has been described, dubbed “3PA”; and has been found to have an association with both SDHB and SDHD mutations [125]. Thus SDH mutations should be suspected in patients presenting with PPGL and a personal or family history of pituitary adenoma.

As in adrenal disease, the secretory pattern for extra-adrenal paragagliomas due to SDH mutations is predominantly dopaminergic; while noradrenergic disease is associated with SDH mutations and, less frequently, VHL/MAX mutations [92]. As with adrenal lesions, immunohistochemical staining for SDHB antibody may be useful [115].

In summary, all patients with extra-adrenal disease should be screened for genetic causes, but screening can be targeted towards the most likely causes.

- Clinical suggestion VHL- Screen VHL gene
- Malignant disease- Screen SDHB, if negative consider SDHD, then SDHC, VHL, MAX
- Head and neck disease- SDHD screening, if negative SDHB/SDHC
- Other extra-adrenal disease- SDHB, if negative consider SDHD, VHL, MAX
- Predominantly noradrenergic/dopaminergic- SDH mutation (if purely noradrenergic consider VHL/MAX).

It is also important to be mindful that studies have suggested that 8–31% of PPGLs which present in an apparently sporadic manner are in fact associated with an underlying germline mutation in one of the aforementioned genes [88, 96, 119, 126–130], and this figure is rising inexorably the more that we understand these tumours. At present the decision to screen genetically (and the guidance available around this) depends on consideration of the likelihood of an underlying condition but also the cost – both financial and time costs – in the screening. As the costs fall and the pickup rate rises it is likely that genetic screening will and should be offered to a wider subgroup of these patients. As many of the genes have limited penetrance the decision points then shift from who to screen to what to do with the results of the screening and the cost / benefit analysis of a surveillance programme in terms of price, incidence and morbidity due to tumours and radiation risk to and anxiety generation for gene-positive family members. A recent study found that 25% of patients with asymptomatic SDHB mutations (identified through relationship to an index case) developed SDHB related tumours 2–6 years after identification of the mutation [131]; the psychological impact of testing on asymptomatic carriers has not yet been assessed. It is known that carriers of genes for other conditions have increased levels of health anxiety and depression [132, 133]; it is essential, therefore, that counselling is carried out prior to genetic testing and that ongoing support is available to the patients.

## Medullary thyroid cancer (MTC)

MTC is a rare tumour, arising from parafollicular, calcitonin producing c-cells of the thyroid gland. It most commonly presents as a painless thyroid mass, frequently with associated adenopathy [134]. The disease is frequently metastatic at presentation [135], and shows poor response to cytotoxic chemotherapy [136].

### In which patients should inherited forms of MTC be suspected?

It is estimated that approximately 25% of MTC is hereditary and associated with germline mutations in the RET (rearranged during transfection) proto-oncogene [137]. Activating mutations of the RET gene are associated with the development of three distinct clinical syndromes: multiple endocrine neoplasia types 2 and 3 (traditionally named MEN2A/MEN2B) and familial medullary thyroid cancer (FMTC) [138–140]. As with MEN1 all are inherited in an autosomal dominant pattern, with varying degrees of penetrance. There has been some debate over the entity of FMTC; which presents with later diagnosis of MTC. In many families in which the diagnosis of FMTC some members of the kindred have gone on to develop phaeochromocytoma leading to some to suggest that FMTC is a lower penetrance form of MEN2A [18].

Inherited tumours are more likely to occur at a younger age [141], and are more frequently multifocal and bilateral [135]. However, some studies have identified patients with RET mutations as late as the eighth decade of life [142–145]. Up to 7% of patients with apparently sporadic disease actually have an underlying RET gene mutation which may only present during the typical age range for sporadic tumour development [143]. Therefore, while older age at diagnosis may suggest that the tumour is more likely to be sporadic, it cannot be assumed.

MEN2 is associated with a 50% risk of developing phaeochromocytoma and 20–30% primary hyperparathyroidism [146] but is associated with MTC in almost all patients [147]. It is associated with a variety of mutations affecting codons 609, 611, 618, 620 and 634 [137]. It is also rarely associated with Cushing’s Disease; the coexistence of hyperparathyroidism and hypercortisolism is associated with both MEN1 and MEN2 [148]. Mutations at codon 634 are by far the most common [149]. MTC is usually the first endocrinopathy to appear in MEN2 [149], with frequency and age of development of hyperparathyroidism or phaeochromocytoma dependent on the RET mutation inherited [150]. Furthermore, the hyperparathyroidism in MEN2A is frequently mild and asymptomatic, and unlikely to be the cause for presentation [151]. Thus, screening for associated conditions is insufficient in out-ruling a genetic cause of MTC. In children with MTC, MEN3 should also be suspected. This is associated with the most aggressive disease and usually presents in childhood. It is associated with a typically marfanoid body habitus and generalised ganglionueromas. In approximately half of patients the mutation has occurred *de novo*. Most commonly associated with mutations in exon 16 (M918 T) and less often exon 15 (A883F) of the RET gene, MEN3 is associated with marfanoid habitus and mucosal neuromas; and phaechromocytoma in 50% of those who survive to adulthood [137]. However, MTC usually develops in infancy before these features are noticed.

Given that inherited MTC can present at any age, and is not universally associated with other features universal screening for genetic mutations in all diagnoses of MTC is recommended [149]. Unlike the situation with MEN1 and the phaeochromocytoma pre-disposing gene mutations there is generally a clear genotype-phenotype correlation between gene mutation and clinical phenotype in MTC. C-Cell hyperplasia, a precursor to MTC can be found in most patients from early childhood. Therefore, in families where the most aggressive mutations are found (M918 T, A883F) a recommendation can be made for prophylactic thyroidectomy as soon as possible and ideally in the first year of life for those found to have the gene mutation in the absence of disease. In milder phenotypes thyroidectomy may delayed until childhood, but in almost all cases should be performed by aged 5 [152]. In older patients, with less aggressive mutations, screening for RET mutations allows for prophylactic thyroidectomy and therefore disease prevention.

In addition, as mentioned earlier half of all patients with MEN2/3 will also develop phaeochromocytoma, with a mean age of presentation of 36 years old, with the phaechromocytoma present by this time MTC is identified in around 40% of cases [137]. It is imperative that phaeochromocytoma be identified before surgery proceeds for MTC, as the morbidity and mortality associated with a hypertensive crisis is significant [153].

## Conclusions

In recent years, our knowledge regarding the genetic mutations underpinning a variety of NETs has expanded rapidly. Traditional DNA sequencing techniques have been largely replaced by “whole-exome sequencing” which allows detailed examination of a gene using next-generation sequencing techniques [100]. This has led to the development of “panels” which can be used in screening for the aforementioned conditions; basic panels include the more common genes, while extended or comprehensive panels look for rarer gene mutations or sequence with a view to detecting any known mutation [100]. As these panels become less expensive and more widely available more questions are raised. For example, should patients who had genetic testing performed several years ago undergo re-testing with the newer techniques? A number of studies have identified gene mutations in patients previously thought to have sporadic MTC or PPGL [88, 117, 126–129, 154, 155]. However, increased screening has in some situations led to increased uncertainty, with mutations of unknown pathogenicity found; some of these in probands with tumours which appear sporadic, with no evidence of disease in family members [156]. This leads to uncertainty regarding correct clinical follow-up for the patient, and family members. Similarly, patients may present with a typical presentation, which is highly suggestive of a syndromic cause of disease, but have a negative genetic test. Currently there are no clear guidelines on how these patients and their families ought to be followed-up. If we decide in these situations to follow up relatives of the proband, we must accept that it is very likely that healthy people will end up having uneccessary imaging/biochemical testing; whereas the opposite also holds true. The number of these patients continues to grow, while in many cases optimal screening programmes have not yet been determined.

The economic cost of screening must also be taken into account; not simply the cost of testing in itself, but the cost of screening family members and providing long term follow up to these likely asymptomatic persons. Many of these conditions have a low penetrance and do not have a clear genotype-phenotype correlation, even within a single family. Thus, the long term economic and psychosocial cost of this testing remains largely unknown, particularly in the case of PPGLs.

The advent of more widely available testing in conjunction with our increased knowledge has also resulted in a change in how patients present. Increasingly we can expect well patients with no history of disease but a positive genetic screening test will be seen in an endocrine clinic. At present screening for NETs and associated conditions has not been standardised; and duration of follow up required remains an unknown.

Cognisant of these difficulties, gene-specific databases have been established in order to catalogue genes implicated in the development of NETs (in particular PPGL) and classify each discovered variant as not pathogenic, unknown significance or likely/definitely pathogenic [100]. Moving forward, more research is needed to determine if phenotype-genotype relationships can be more reliably classified so that patients may be given a more adequate estimation of risk. We also need to remain conscious of the possible psychological effects of screening, not only on patients, but on their relatives.

Clinicians should be encouraged to ensure that patients and their families are managed in centres with relevant expertise. For example, in Europe, there is a list of approved centres of excellence for the management of NETs, although unfortunately there is not a centre in every European country at present [157]. Patients and their families may also benefit from access to peer support and there are a number of organisations which they can be referred to across the world such as PheoParatroopers [158], NET Patient Foundation [159] and the Association for Multiple Endocrine Neoplasia Disorders which has a European and US branch [160]. All of these organisations provide accessible literature for patients, patient information days and often a helpline or counselling service. The NET Alliance contains a list of patient advocacy groups from across the globe [161].

Genetic counselling prior to screening for a variety of conditions, in a dedicated genetics clinic has been shown to improve patient satisfaction with care, and improve the accuracy of patient perception of future risk following their diagnosis [162, 163]. To our knowledge, no study has been undertaken specifically in patients with NETs but we would suggest the same principles are likely to hold true. We would therefore suggest that patients in whom you suspect a likely germline genetic mutation be referred to a dedicated genetics clinic in an experienced centre prior to testing.
